# PERFORMANCE ON COGNITIVE FUNCTIONING RELATED TO SUCCESSFUL AGING

**DOI:** 10.1093/geroni/igz038.2426

**Published:** 2019-11-08

**Authors:** Neyda Ma Mendoza Ruvalcaba, Elva Dolores Arias Merino, Maria Elena Flores Villavicencio, Melina Rodriguez Díaz

**Affiliations:** 1 Universidad de Guadalajara CUTONALA, Guadalajara, Mexico, Mexico; 2 Universidad de Guadalajara CUCS, Guadalajara, Mexico, Mexico; 3 University of Guadalajara CUCS, Guadalajara, Mexico, Mexico

## Abstract

Introduction The cognitive functioning, as a general measure, is a criterion commonly used to define and operationalize successful aging. (Project-Conacyt-256589) The aim of this study is to analyze cognitive function and its relationship with the successful aging in older adults. Methods Population based, random sample included n=401 community-dwelling older adults 60-years and older (mean age=72.51,SD=8.11 years,59.4% women). Cognitive functioning was assessed by a comprehensive battery including working memory(Digit Span Backward WAIS-IV), episodic memory, metamemory(self-report), processing speed(Symbol Digit WAIS-IV), attention(TMT-A), executive functioning(TMT-B), learning potential(RAVLT), language(FAS), visuospatial skills(Block Design WAIS-IV). Successful aging was operationalized in accordance with Rowe & Kahn definition (no important disease, no disability, physical functioning, cognitive functioning, and being actively engaged). Sociodemographic and health data were also asked. Data were analyzed in SPSSv24. Results In total 11.2% were successful agers and 11.4% had Mild Cognitive impairment. Global cognitive functioning was significantly related to the achievement of successful aging criteria. Specifically, the more successful agers showed a significant (p′s<.05) better performance on learning potential, working memory, metamemory, processing speed and attention. Executive functions were not related to successful aging criteria. None cognitive domain was related to the being actively engaged criteria. Better visuospatial skills were showed in older adults meeting the criteria of being free of disability and high physical functioning. Conclusion Knowledge generated by this study reveals the role of specific domains of cognitive functioning in successful aging, and sets a scenario to promote successful aging, through alternatives centered in the improvement of cognition in the older adults.

